# Supplementary material for: Real-time, noise and drift resilient formaldehyde sensing at room temperature with aerogel filaments
Source: Sci Adv. 2024 Feb 9;10(6):eadk6856. doi: 10.1126/sciadv.adk6856 (PMC10857368; doi:10.1126/sciadv.adk6856)
Supplement: Supplementary file 1 — Supplementary Text Figs. S1 to S15 Tables S1 to S3 Legend for data S1 References [file sciadv.adk6856_sm.pdf]

Science Advances
AAAS

Supplementary Materials for

Real-time, noise and drift resilient formaldehyde sensing at room temperature with aerogel filaments

Zhuo Chen *et al.*

Corresponding author: Tawfique Hasan, th270@cam.ac.uk

Sci. Adv. **10**, eadk6856 (2024)
DOI: 10.1126/sciadv.adk6856

The PDF file includes:

Supplementary Text
Figs. S1 to S15
Tables S1 to S3
Legend for data S1
References

Other Supplementary Material for this manuscript includes the following:

Data S1

Supplementary Text

Diffusion coefficient of formaldehyde in aerogels

The structural dependence of the gas sensor response can be understood by studying the gas-diffusion profile inside filament and film-like aerogels. The dominating diffusion mechanism inside aerogels is molecular diffusion instead of Knudsen diffusion as their pores' diameters are in the order of μm. The gas molecules have mean-free-path much smaller than the pore size and their diffusion is mainly driven by the concentration gradient and less affected by collisions with the walls. We first calculate the diffusion coefficient D of trace-level formaldehyde gas in the air following Fuller's method (*45*). In general, the expression for the diffusion coefficient of trace gas A in carrier gas B is as follows:

$$D_{AB} = \frac{0.00143T^{1.75}}{P \times \sqrt{M_{AB}} \times \left[\sqrt[3]{(\sum_n V_i)_A} + \sqrt[3]{(\sum_n V_i)_B}\right]^3} \tag{S1}$$

where T is temperature in K, P is pressure in bar, $\sum_n V_i$ is molecular diffusion volume calculated by summing the constituent atomic diffusion volumes V_i, and M_{AB} is the reduced mass of the molecular pair A and B, given by

$$M_{AB} = \frac{2}{\frac{1}{M_A} + \frac{1}{M_B}} \tag{S2}$$

where M_A and M_B are the molar masses in g mol^{-1} of A and B, respectively. The diffusion volumes for formaldehyde and air are found to be 26.63 and 19.7, respectively (*45*, *46*), whereas the reduced mass of such pair is calculated as 29.44 g mol^{-1}. Thus, the diffusion coefficient of formaldehyde in air is calculated to be 1.67×10^{-5} m^2 s^{-1} at room temperature and atmospheric pressure.

Concentration profile in filament and film-like aerogels

The concentration profile inside gas sensors can be approximated by combining Fick's law of diffusion and Arrhenius' law (*39*, *47*). The target gas molecules are diffusing into aerogel materials due to a concentration gradient between the surface concentration C_S and the concentration inside C. Meanwhile, gas molecules react with the oxygen ions absorbed on the metal oxide materials and are simultaneously consumed. For thin-film-like aerogels, the diffusion equation taking into consideration of the surface reaction is described below:

$$\frac{\partial C}{\partial t} = D\frac{\partial^2 C}{\partial x^2} - kC \tag{S3}$$

where x is the distance from the bottom of the material, and k is the surface reaction rate given by

$$k \propto exp\left(\frac{-E_\mathrm{a}}{RT}\right) \tag{S4}$$

where E_a is the activation energy and R is the molar gas constant. Considering the steady state when $\partial C/\partial t = 0$, the general solution is expressed as:

$$C = c_1 e^{\sqrt{\frac{k}{D}}x} + c_2 e^{-\sqrt{\frac{k}{D}}x} \tag{S5}$$

Finally, we apply the boundary conditions, $C = C_S$ at the aerogel surface ($x = L$) and $\partial C/\partial x = 0$ at the bottom ($x = 0$), the following equation for thin-films can be obtained:

$$C = C_s \frac{cosh\left(\sqrt{\frac{k}{D}}x\right)}{cosh\left(\sqrt{\frac{k}{D}}L\right)} \tag{S6}$$

Next, we derive the concentration profile expression inside filament structured aerogels from the diffusion equation for a system in cylindrical coordinates as below:

$$D_{11}\left[\frac{1}{r}\frac{\partial}{\partial r}\left(r\frac{\partial C}{\partial r}\right) + \frac{1}{r}\frac{\partial^2 C}{\partial \phi^2}\right] + D_{33}\frac{\partial^2 C}{\partial z^2} - kC = \frac{\partial C}{\partial t} \tag{S7}$$

We then assume that the diffusion is anisotropic and gas molecules only diffuse radially inwards from the surface but not along the longitudinal direction of the filament, we can then explore cylindrical symmetry and simplify Supplementary equation S7 to

$$D\frac{1}{r}\frac{\partial}{\partial r}\left(r\frac{\partial C}{\partial r}\right) - kC = \frac{\partial C}{\partial t} \tag{S8}$$

or equivalently

$$D\frac{\partial^2 C}{\partial r^2} + D\frac{1}{r}\frac{\partial C}{\partial r} - kC = \frac{\partial C}{\partial t} \tag{S9}$$

where r is the distance from the center of the filament. At equilibrium when $\partial C/\partial t = 0$, this can be further simplified to

$$r^2\frac{\partial^2 C}{\partial r^2} + r\frac{\partial C}{\partial r} - \frac{k}{D}Cr^2 = 0 \tag{S10}$$

which is the modified Bessel differential equation with a general solution:

$$C = c_1 I_0\left(\sqrt{\frac{k}{D}}r\right) + c_2 K_0\left(\sqrt{\frac{k}{D}}r\right) \tag{S11}$$

where I_0 and K_0 are modified Bessel function of the first kind and second kind, respectively. With the boundary conditions, $C = C_S$ at the aerogel surface ($r = R$) and $\partial C/\partial r = 0$ at the bottom ($r = 0$), the following equation for filaments can be obtained:

$$C = C_s \frac{I_0\left(\sqrt{\frac{k}{D}}r\right)}{I_0\left(\sqrt{\frac{k}{D}}R\right)} \tag{S12}$$

We then calculate the concentration profiles for filament-structured aerogels with diameter of 250, 400, and 600 μm, as well as film-like aerogels with thickness of 250, 400, and 600 μm. The surface reaction rate k is set to be 200 s^{-1}.

Baseline drift resilience investigation

Gas sensors based on metal oxide semiconductors (MOS) generally have long-term stability and baseline drift issues due to various reasons, such as slow equilibration of defects, poisoning by strongly absorbed gas species, thermo-mechanical degradation, environmental change, etc. (*20*) Possible measures to mitigate the defect-related baseline drift are proper aging of the fabricated

sensors to accelerate defects equilibration and doping of metal impurities to stabilise the density of oxygen vacancies (*21*). The absorption-related baseline drift can be alleviated partially by applying heating pulses for MOS sensors working near room temperature to accelerate the gas desorption. Appropriate design and selection of MEMS microheater substrates can extend the heater lifetime and thus reduce thermo-mechanical degradation. Temperature and humidity changes during daily use can be addressed with integrated temperature and humidity sensors and compensated through pre-trained models. However, these techniques require intensive materials and device optimisation or large amounts of data for intensive model training. More critically, for the task of gas classification, any undealt baseline drift will result in overlapping of data clusters that represent different gas species in the feature and render the pre-trained model unstable and ineffective. Thus, there is a genuine interest in developing classification algorithms that are inherently resilient to the baseline drift to extend the lifetime of MOS-based gas sensors cost-effectively.

To investigate the baseline-drift resilience of our dynamic-feature-based classification algorithms, we first model the baseline drift as an additive drift process (*48*) as below:

$$R_{\mathrm{d}}(t) = R(t) \times (1 + \alpha t) \tag{S13}$$

where $R(t)$ is the resistance without any drift, α is the drift coefficient, and αt is the relative drift at certain time. We denote the stabilised sensor resistance in the air without any drift at $t = 0$ as R_0, and calculate the nominal sensor response with drift as below:

$$Rsp_{\mathrm{d-nominal}}(t) = \frac{R_0 - R_{\mathrm{d}}(t)}{R_0} = 1 - \frac{R(t) \times (1 + \alpha t)}{R_0} \tag{S14}$$

The nominal response curves with baseline drifts of different α are plotted in Fig. S1A. A negative α will result in smaller resistance but larger response values with time, whereas a positive α will lead to the increase of resistance but decrease of response. Here the α value is in the order of percentage per hour for the illustration purpose only, which is much larger than most commercial gas sensors that drift with a time scale of days or months.

Notably, if the response values are calculated with respect to the most recent resistance value in ambient air $R_a(t)$ instead of R_0, we can define the true response with drift as below:

$$\begin{aligned} Rsp_{\mathrm{d-true}}(t + \Delta t) &= \frac{R_a(t) - R_{\mathrm{d}}(t + \Delta t)}{R_a(t)} \\ &= \frac{R_0 \times (1 + \alpha t) - R(t + \Delta t) \times (1 + \alpha t + \alpha \Delta t)}{R_0 \times (1 + \alpha t)} \end{aligned} \tag{S15}$$

if α is sufficiently small or the drift is slow enough, this can be simplified to:

$$Rsp_{\mathrm{d-true}}(t + \Delta t) = \frac{R_0 - R(t + \Delta t)}{R_0} \tag{S16}$$

which is exactly the response without any drift. In other words, $Rsp_{\mathrm{d-true}}$ will not change with the baseline drift and can be readily used for stable classification. In reality, $Rsp_{\mathrm{d-true}}$ may be calculated by calibrating the sensor baseline resistance with clean air regularly during the field operation or designing self-calibration algorithms to identify and select a new baseline resistance in the intermittence of gas exposure. However, these measures still require considerable maintenance efforts, whereas $Rsp_{\mathrm{d-nominal}}$ can be easily calculated using the initial R_0 value and the real-time resistance readout $R_{\mathrm{d}}(t)$. We will thus focus on using the nominal response value

and assess the baseline drift resilience of the dynamic-feature-based algorithms.

We then assume that the baseline drift is slow enough such that the resistance values can be treated as if there is no drift within the timeframe of target exposure or measurement, and the drift only becomes prominent when compared to the initial resistance. The corresponding nominal response curves with positive α values are shown in Fig. S1B, where the response values drift by 10 %, 20 %, and 30 % with respect to R_0. For the response curve with a larger baseline drift, the response values are smaller while the difference between the response value in ambient air and in target gas becomes larger. On the other hand, the nominal response curves with more negative α values (Fig. S1C) show the opposite trends by having larger response values and smaller differences between the response in ambient air and target gas. In other words, the values and the response curves' shapes are both changing with the drift. It is thus impossible to directly use the nominal response values for classifying different gas species but instead requires the extraction of drift-resilient features.

Following the expression of the nominal response and the assumption of slow baseline drift, we can calculate the rate-of-change (RoC) value with baseline drift shown as below:

$$\begin{aligned} RoC_d(\Delta t) &= \frac{Rsp_{\text{d-nominal}}(t+\Delta t) - Rsp_{\text{d-nominal}}(t)}{\Delta t} \\ &= \frac{1}{\Delta t} \times \left[\frac{R_0 - R(t+\Delta t) \times (1+\alpha t')}{R_0} - \frac{R_0 - R(t) \times (1+\alpha t')}{R_0}\right] \\ &= (1+\alpha t') \times \frac{1}{\Delta t} \times \frac{R(t) - R(t+\Delta t)}{R_0} \end{aligned} \tag{S17}$$

Meanwhile, the RoC value without any baseline drift is expressed as below:

$$\begin{aligned} RoC(\Delta t) &= \frac{Rsp(t+\Delta t) - Rsp(t)}{\Delta t} \\ &= \frac{1}{\Delta t} \times \left[\frac{R_0 - R(t+\Delta t)}{R_0} - \frac{R_0 - R(t)}{R_0}\right] \\ &= \frac{1}{\Delta t} \times \frac{R(t) - R(t+\Delta t)}{R_0} \end{aligned} \tag{S18}$$

Thus, we have the following relation:

$$RoC_d(\Delta t) = (1+\alpha t') \times RoC \tag{S19}$$

Note that $1+\alpha t'$ remains constant during the timeframe of target gas exposure and measurement. Once the RoC features are normalised, this constant term will be eliminated such that $RoC_d(\Delta t)$ is equivalent to $RoC(\Delta t)$. Any classifiers trained with the normalised RoC features are thus inherently resilient to any additive baseline drift.

Similarly, we can express the discrete Fourier transform (DFT) of the nominal response values in the presence of baseline drift as follows:

$$\begin{aligned} X_{\text{d}}(f) &= \sum_{t=0}^{N-1} Rsp_{\text{d-nominal}}(t) \times e^{-\frac{i2\pi}{N}ft} \\ &= \sum_{t=0}^{N-1} \frac{R_0 - R(t) \times (1+\alpha t')}{R_0} \times e^{-\frac{i2\pi}{N}ft} \end{aligned}$$

$$= \sum_{t=0}^{N-1} e^{-\frac{i2\pi}{N}ft} - (1 + \alpha t') \times \sum_{t=0}^{N-1} \frac{R(t)}{R_0} \times e^{-\frac{i2\pi}{N}ft} \quad \text{(S20)}$$

In comparison, the complex DFT values without any baseline drift are calculated as

$$X(f) = \sum_{t=0}^{N-1} Rsp(t) \times e^{-\frac{i2\pi}{N}ft}$$
$$= \sum_{t=0}^{N-1} e^{-\frac{i2\pi}{N}ft} - \sum_{t=0}^{N-1} \frac{R(t)}{R_0} \times e^{-\frac{i2\pi}{N}ft} \quad \text{(S21)}$$

Then we have the following relation:

$$X_{\mathrm{d}}(f) = (1 + \alpha t') \times X(f) - \alpha t' \times \sum_{t=0}^{N-1} e^{-\frac{i2\pi}{N}ft}$$
$$= \begin{cases} (1 + \alpha t') \times X(f) - \alpha t' \times N & for\, f = 0 \\ (1 + \alpha t') \times X(f) & for\, f > 0 \end{cases} \quad \text{(S22)}$$

where the $\sum_{t=0}^{N-1} e^{-\frac{i2\pi}{N}ft}$ term is recued to zero due to symmetry for non-zero frequencies. This implies that for any $f > 0$, the complex DFT values with baseline drift only differ from that without drift by a factor of $1 + \alpha t'$, which will again be eliminated by the subsequent normalisation process. In contrast, the DFT value with baseline drift at the fundamental frequency $f = 0$ has a persistent $-\alpha t' \times N$ term and are not equivalent to that without drift after normalization. The corresponding DFT magnitudes $M(f)$ follow the same rules as above, proving that normalised high-order DFT magnitudes are inherently resilient to any additive baseline drift and classification based on these features are stable regardless of the drift values.

Influence of noise on the baseline drift resilience

The influence of noise on the baseline drift can be studied by including a simulated Gaussian noise in the baseline drift model, and the nominal response with drift and noise is expressed as:

$$Rsp_{\mathrm{d,n-norminal}}(t) = \frac{R_0 - R_{\mathrm{d}}(t)}{R_0} + s \times N(0,1) \quad \text{(S23)}$$

where $N(0,1)$ is the standard normal distribution with zero mean and unity standard deviation, and $s \times N(0,1)$ represents the Gaussian noise with a standard deviation of s which can also be written as $N(0, s^2)$. Note that the value of this Gaussian noise is varying at different time t and will be denoted as $s \times n(t)$.

The RoC values extracted from signals with both baseline drift and noise are expressed as below:

$$RoC_{d,n}(\Delta t) = \frac{Rsp_{\mathrm{d,n-nominal}}(t + \Delta t) - Rsp_{\mathrm{d,n-nominal}}(t)}{\Delta t}$$
$$= (1 + \alpha t') \times RoC + s \times \frac{n(t + \Delta t) - n(t)}{\Delta t} \quad \text{(S24)}$$

After normalization, the noise term $s \times \frac{n(t+\Delta t)-n(t)}{\Delta t}$ will be scaled down by an approximate factor of $1 + \alpha t'$. Thus a positive baseline drift effectively reduces the standard deviation of noise and its influence on normalised RoC features, resulting in an improved prediction accuracy as shown

in Fig. 5G. On the other hand, a negative baseline drift amplifies the influence of noise and leads to less accurate classification. This drift dependence of the classification accuracy is more prominent when the noise standard deviation in the response signals is higher.

Similarly, the DFT values extracted from signals with both baseline drift and noise are expressed as below:

$$X_{\mathrm{d,n}}(f) = \sum_{t=0}^{N-1} Rsp_{\mathrm{d,n-nominal}}(t) \times e^{-\frac{i2\pi}{N}ft}$$
$$= \begin{cases} (1+\alpha t') \times X(f) - \alpha t' \times N + s \times \sum_{t=0}^{N-1} n(t) & for\, f = 0 \\ (1+\alpha t') \times X(f) + s \times \sum_{t=0}^{N-1} n(t) \times e^{-\frac{i2\pi}{N}ft} & for\, f > 0 \end{cases} \quad \text{(S25)}$$

Following the same argument, we can conclude that a positive drift can increase the signal-to-noise ratio in the normalised DFT magnitudes of high-order frequencies which contributes to a small improvement in the classification efficiency as shown in Fig. 5I.

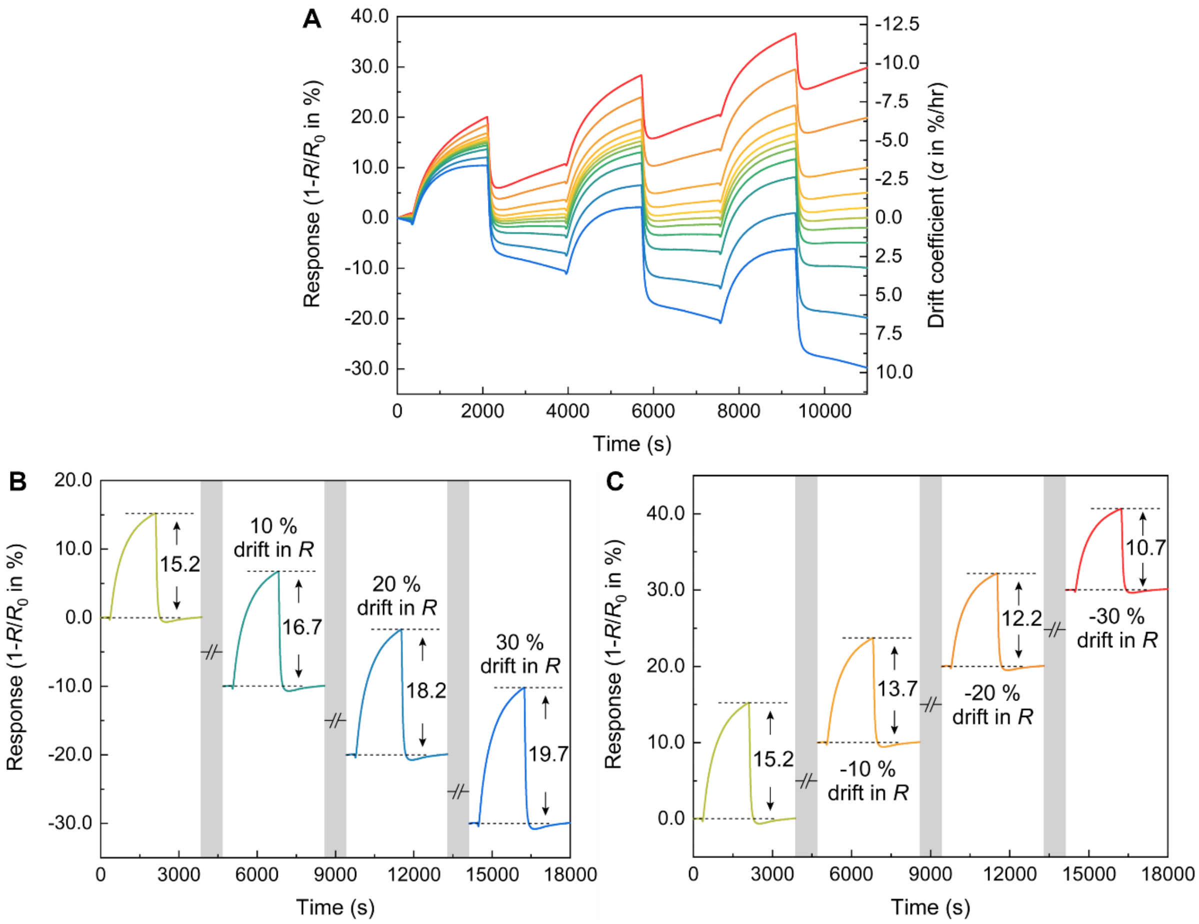

Fig. S1. Simulation of additive baseline drift.

(**A**) Continuous drift of response curves with different drift coefficients. (**B**) Response curves due to slow and positive drift of resistance at different times. The time axis is not to scale. (**C**) Response curves due to slow and negative drift of resistance at different times. The time axis is not to scale.

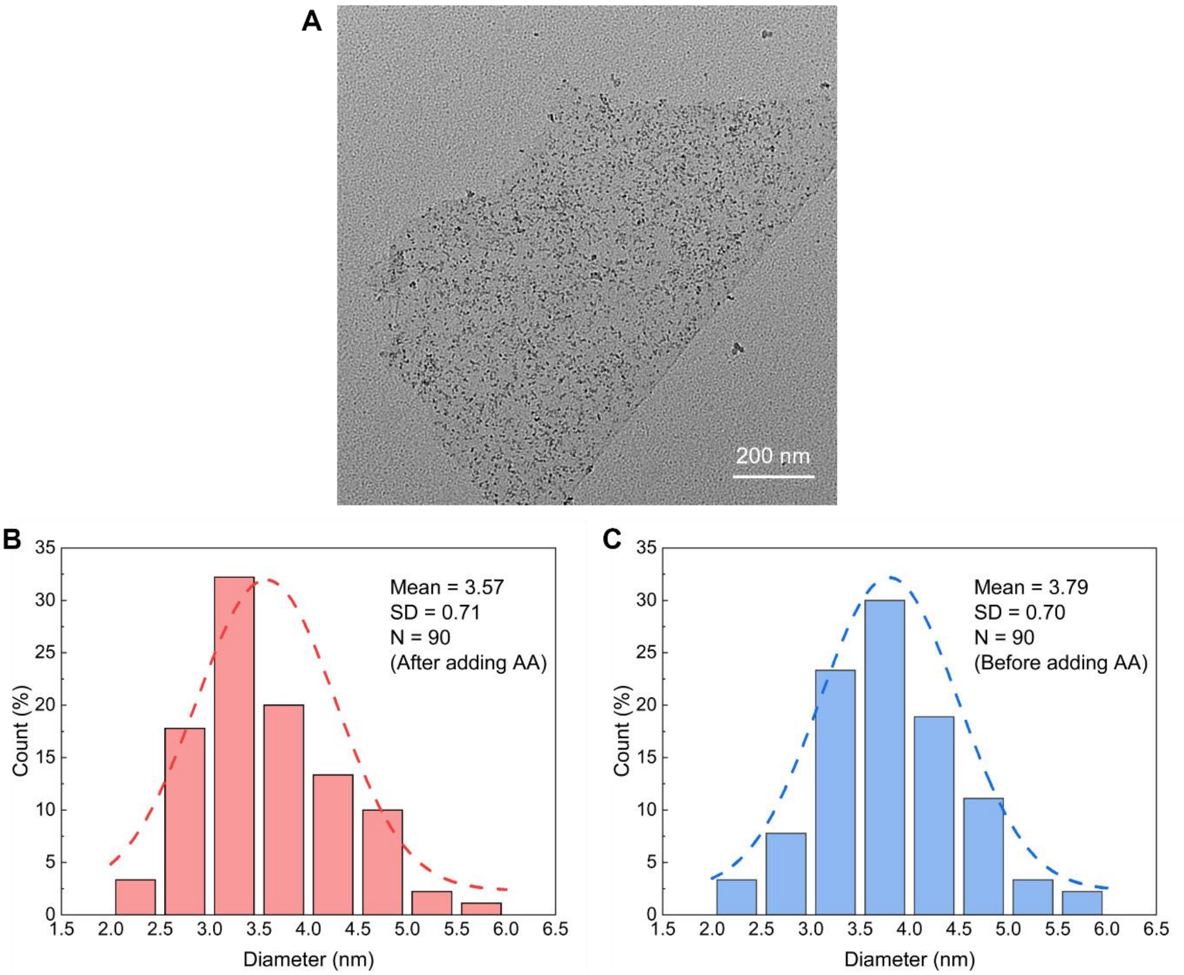

Fig. S2. Particle size distribution of synthesized SnO2 QDs on GO based on transmission electron microscopy (TEM) images.

(**A**) TEM image showing the uniform distribution of SnO_2 QDs on a graphene sheet. (**B**) Particle size distribution of SnO_2 QDs before the addition of ascorbic acid (AA). (**C**) Particle size distribution of SnO_2 QDs after the addition of AA. The total number of counts is 90 for both distributions.

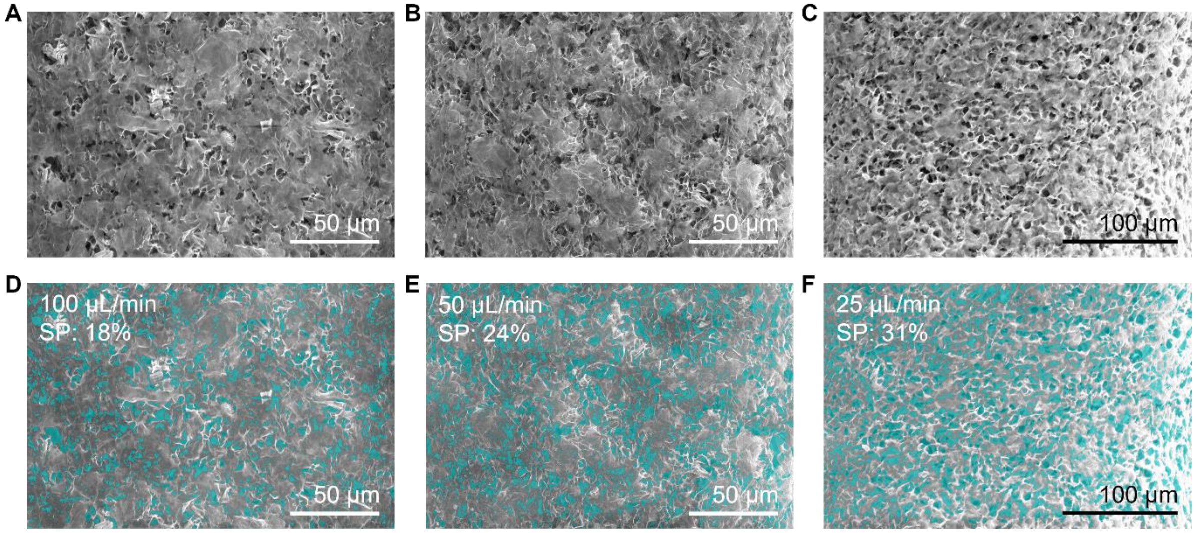

Fig. S3. Scanning electron microscope (SEM) characterizations of aerogels with different surface porosities.

(**A-C**) SEM images of aerogels with surface porosities of 18 %, 24 %, and 31 %, respectively. (**D-F**) Identification of pores' area marked in cyan colour through image processing for aerogels with surface porosities of 18 %, 24 %, and 31 %, respectively.

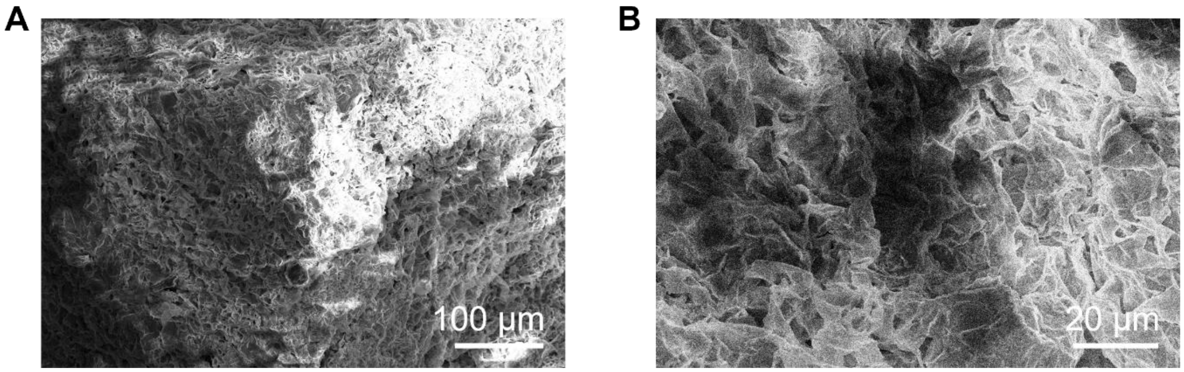

Fig. S4. SEM characterization of aerogels directly dried after being washed with ethanol, showing closed surface with no pores.

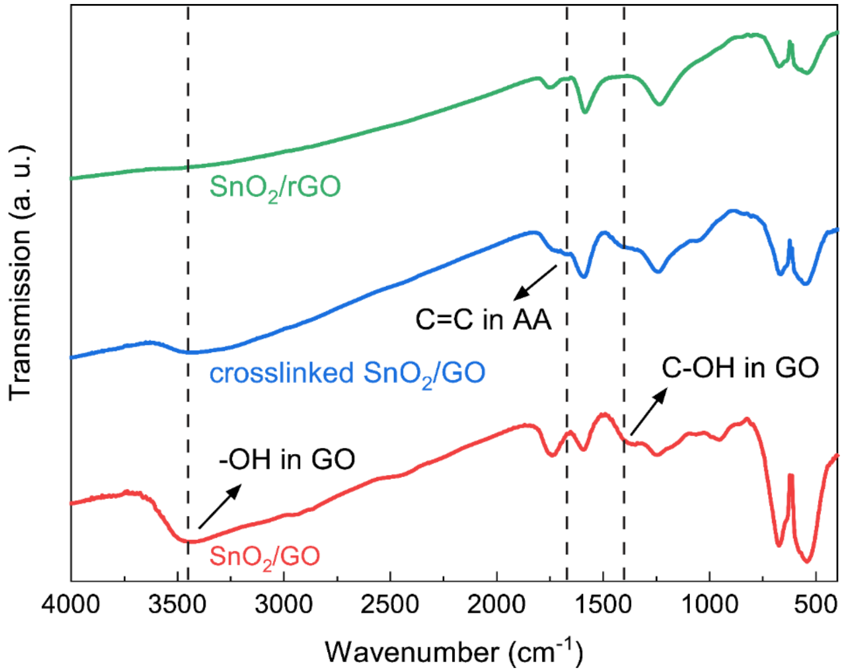

Fig. S5. Transmission Fourier-transform infrared (FTIR) spectra of aerogels.

The red curve is for freeze-casted SnO_2/GO aerogel without any additives or washing. The blue curve is for freeze-casted SnO_2/GO aerogel crosslinked by ascorbic acid (AA), showing weaker -OH and C-OH peaks of GO and C=C peak of AA. The green curve is for freeze-casted SnO_2/GO aerogel crosslinked by AA, washed by ethanol and hexane, and annealed, showing disappearance of relevant peaks of GO and AA. None of these spectra show the peaks of 6-aminohexanoic acid (AHA), indicating that AHA is fully removed after the centrifugal washing process after hydrothermal synthesis.

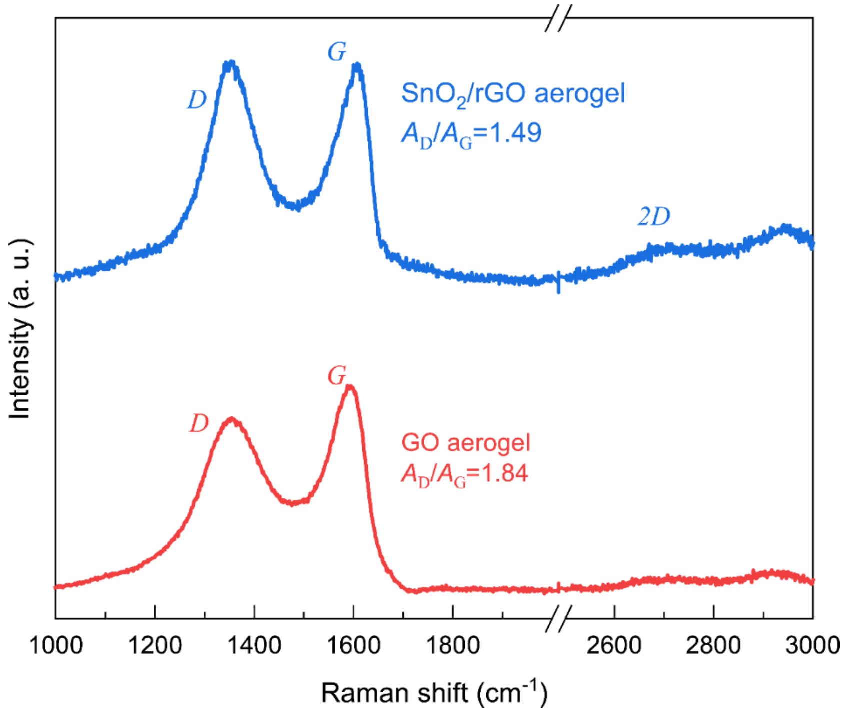

Fig. S6. Raman spectra of aerogels.

The red curve is for freeze-casted SnO_2/GO aerogel, and the blue curve is for aerogel that has been washed and annealed. The excitation wavelength is 532 nm.

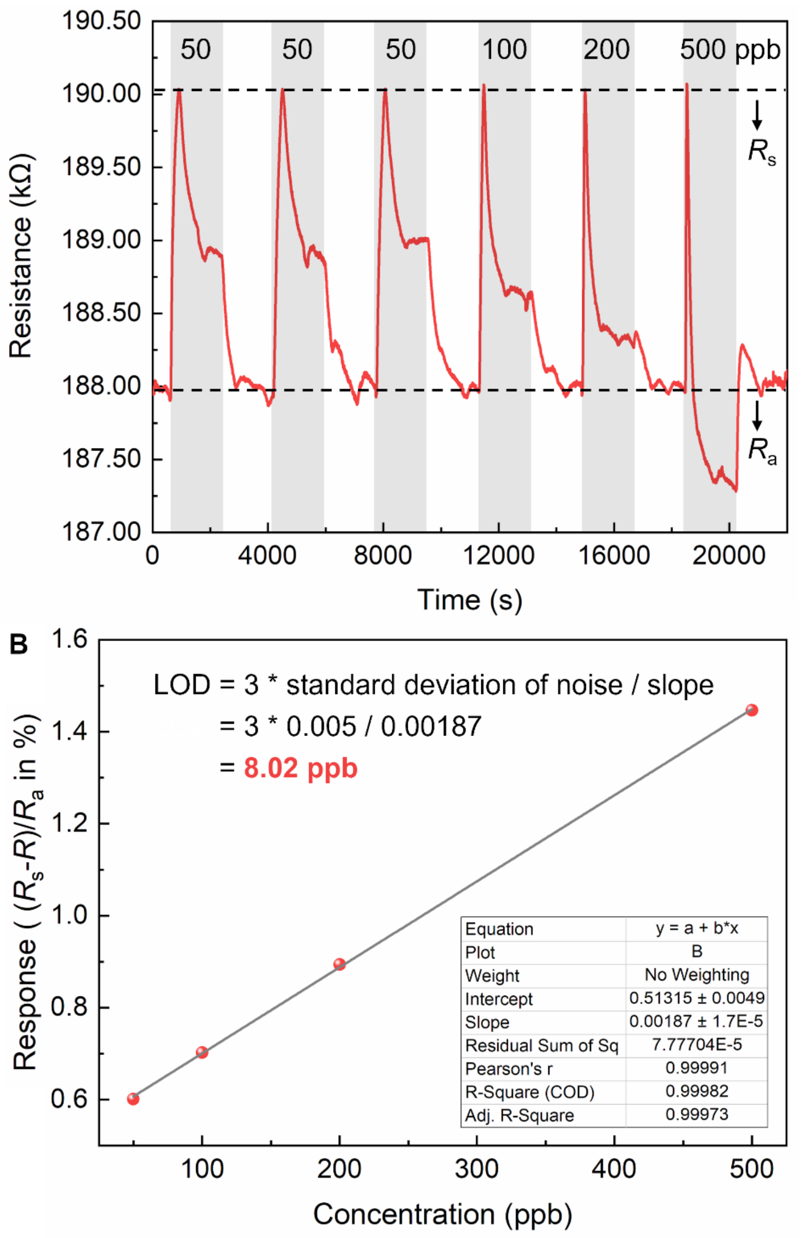

Fig. S7. Sensing measurement of the champion filament-structured aerogel sensor and theoretical limit of detection (LOD) estimation.

(**A**) Transient curves of ultralow concentration gas sensing performance towards 50-500 ppb formaldehyde based on the champion sensor with 250 μm filament diameter, 31 % surface porosity (SP) and Cu doping. The stable resistance in air is denoted as R_a, and the transient and repeatable high resistance value during the initial stage of gas exposure due to the p-type copper oxide at the quantum dots' surface is denoted as R_s. As the concentration of CH_2O inside the aerogel increases due to diffusion, the sensor resistance decreases, showing n-type response by the dominating SnO_2 material. (**B**) LOD estimation from the concentration-response regression curve. In order to keep the response value positive and recognise the repeatable and sensitive response of the p-type surface dopant CuO at initial exposure, the low-concentration response is calculated by dividing the difference between R_s and the end of exposure resistance R, $R_s - R$, by R_a. For high-concentration response, $R_s - R$ can be approximated by $R_a - R$ as used in the main text.

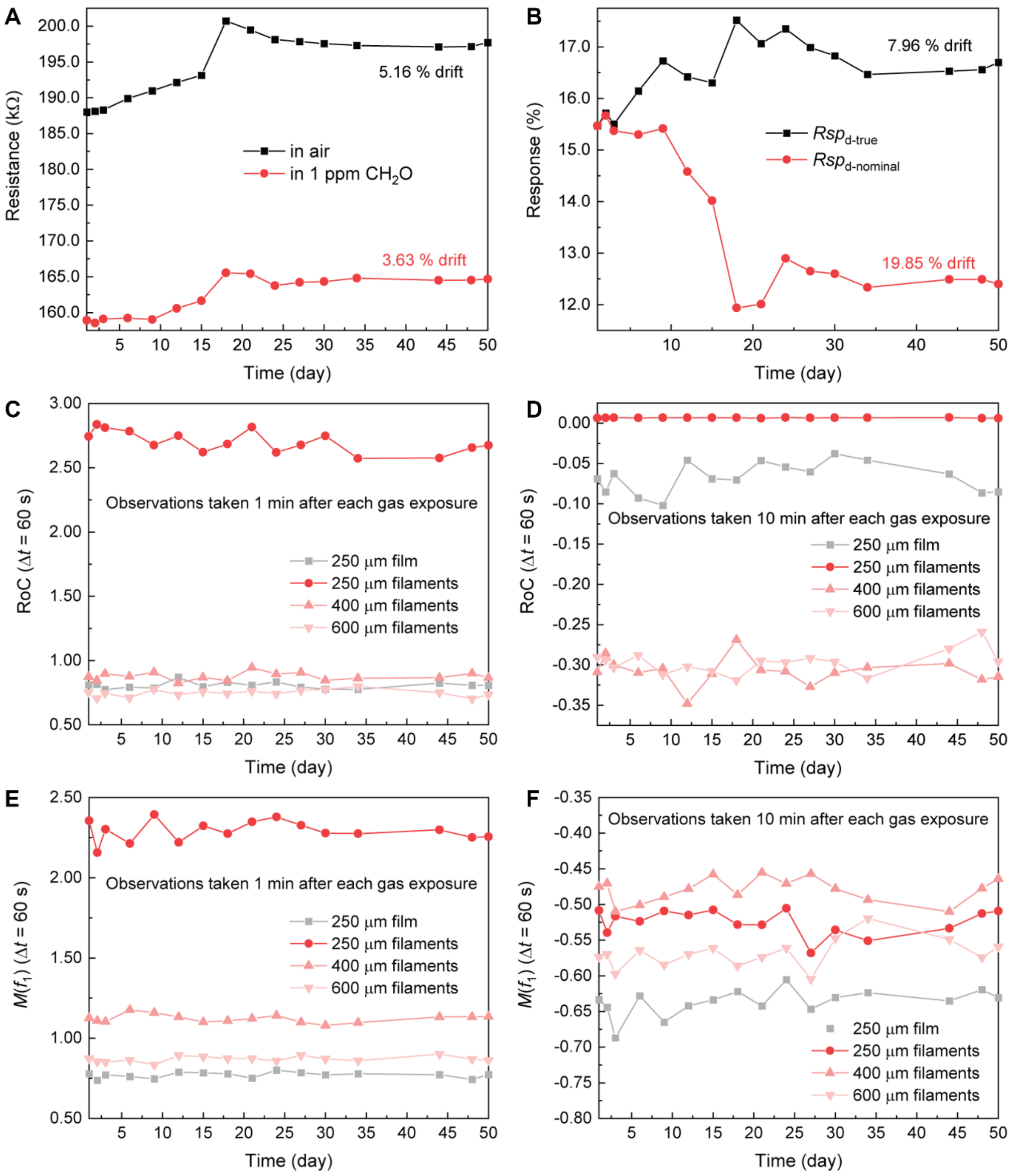

Fig. S8. Long-term stability test of aerogel gas sensor.

(**A**) The long-term stability of the champion sensor with 250 μm filament diameter, 31 % surface porosity (SP) and Cu doping, showing the trends of resistance change in dry air and in 1 ppm CH_2O. (**B**) Trends of true response and nominal response within the 50-day period. Long-term stability of the extracted RoC values based on the structurally multiplexed array with 60 s observation window taken (**C**) 1 min and (**D**) 10 min after gas exposure. Long-term stability of the extracted DFT $M(f_1)$ values based on the structurally multiplexed array with 60 s observation window taken (**E**) 1 min and (**F**) 10 min after gas exposure.

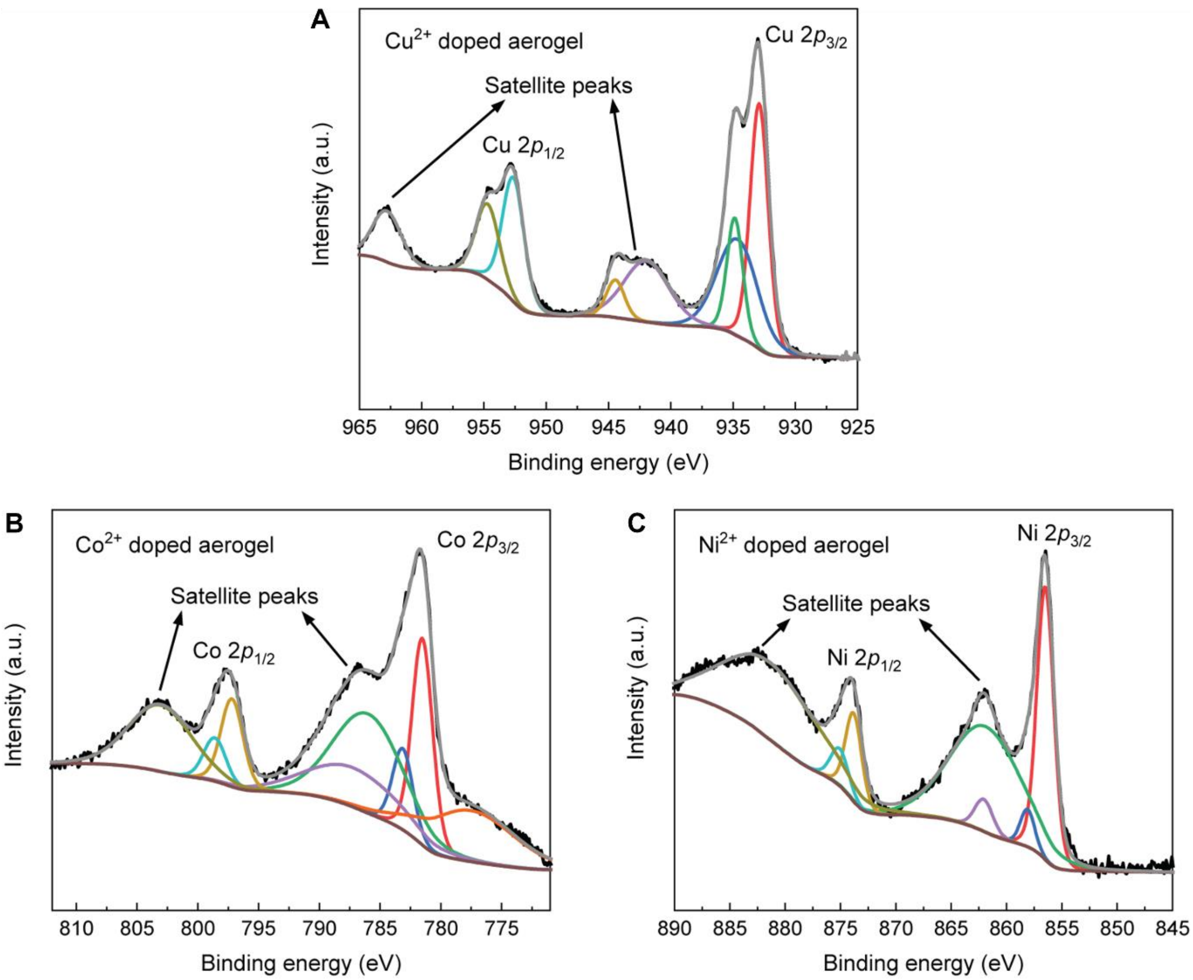

Fig. S9. XPS characterisation of aerogels doped with different metal ions.

XPS spectra of (**A**) Cu^{2+}, (**B**) Co^{2+}, (**C**) Ni^{2+} doped aerogels. The corresponding characteristics peaks and satellite peaks confirm the successful doping of different metal states into the aerogels. Specifically, for Cu^{2+} doped aerogels, the satellite peaks at 944.4 eV and 941.9 eV correspond to the Cu^{2+} state in CuO whereas the peak at 932.9 eV corresponds to the Cu^{+} state in Cu_2O (*49*). For Co^{2+} doped aerogels, the satellite peaks at 783.1 eV, 785.9 eV and 787.2 eV reveal the Co^{2+} state in CoO whereas the peak at 777.5 eV suggests the existence of Co^{3+} state after annealing (*50*). Finally, for Ni^{2+} doped aerogels, the strong peak at 856.5 eV suggests that the Ni^{3+} state is dominating compared to the Ni^{2+} state whose characteristic peak at 854 eV is undiscernible (*51*). All sample data were recorded at a pressure below 10^{-8} Torr and a room temperature of 294 K. Peaks were fitted with a Shirley background prior to component analysis.

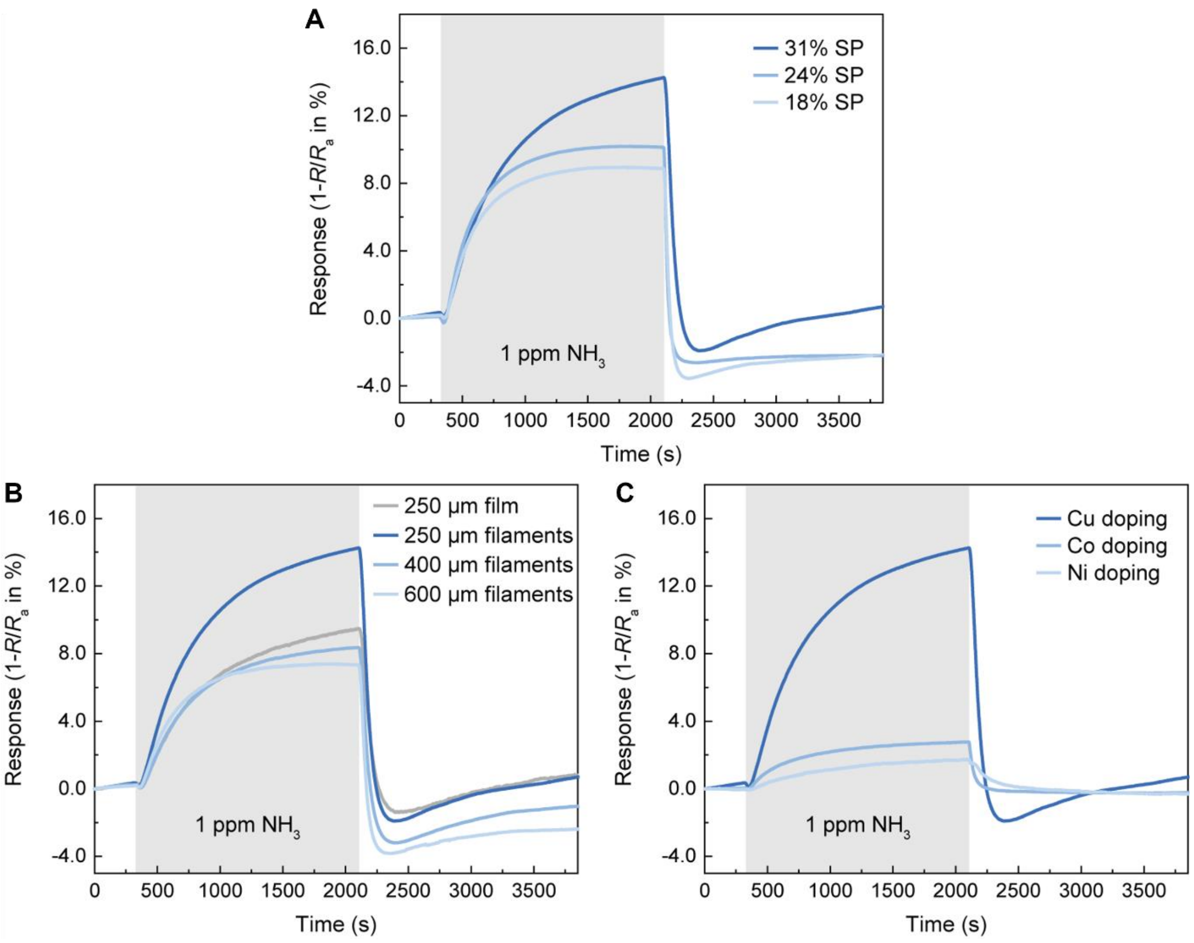

Fig. S10. Room temperature gas sensing characteristics of aerogels towards 1 ppm NH_3. (**A**) Response curves of filament-structured aerogel sensors with different surface porosities (SP). (**B**) Response curves of filament-structured aerogel sensors with different filament diameters and one film-like aerogel sensor. (**C**) Response curves of filament-structured aerogel sensors with different dopants.

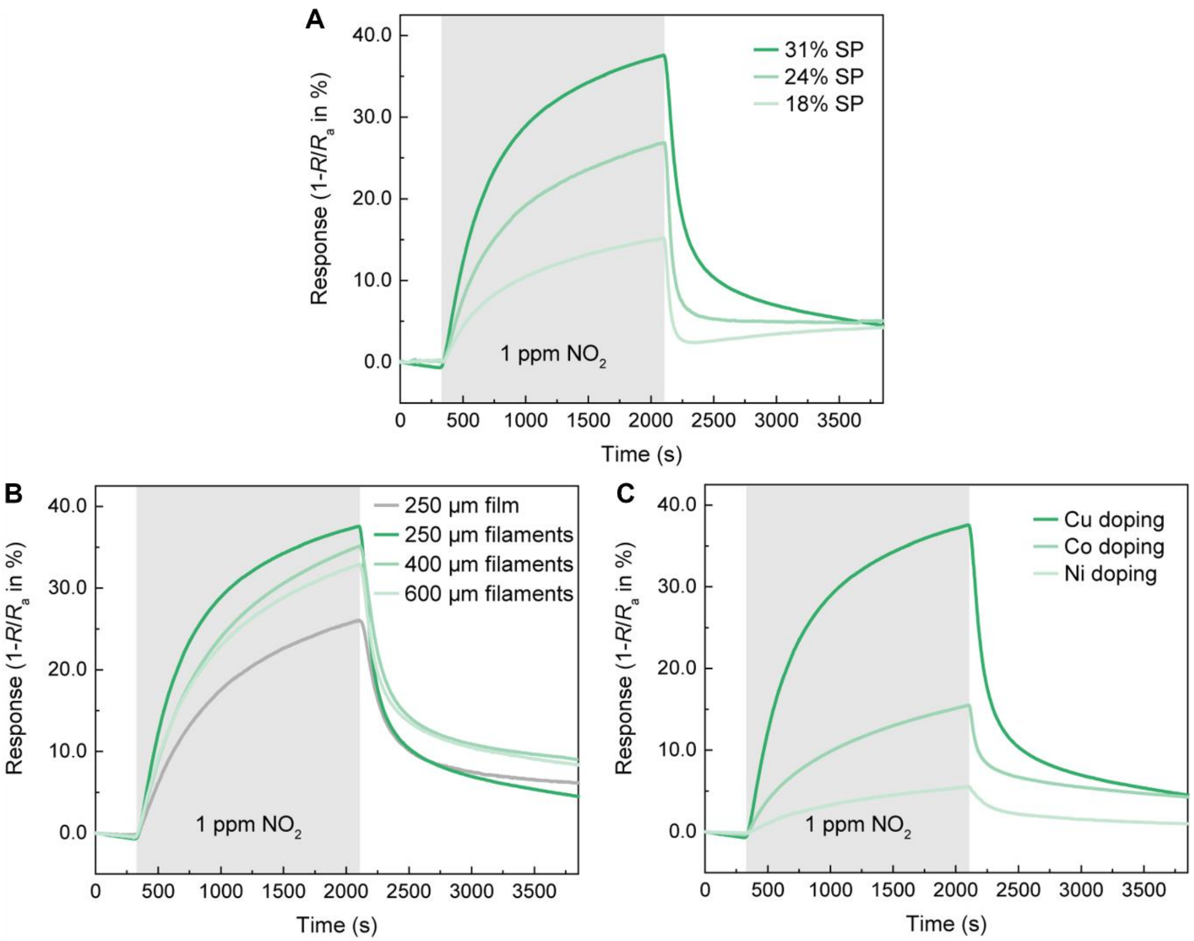

Fig. S11. Room temperature gas sensing characteristics of aerogels towards 1 ppm NO_2.

(**A**) Response curves of filament-structured aerogel sensors with different surface porosities (SP). (**B**) Response curves of filament-structured aerogel sensors with different filament diameters and one film-like aerogel sensor. (**C**) Response curves of filament-structured aerogel sensors with different dopants.

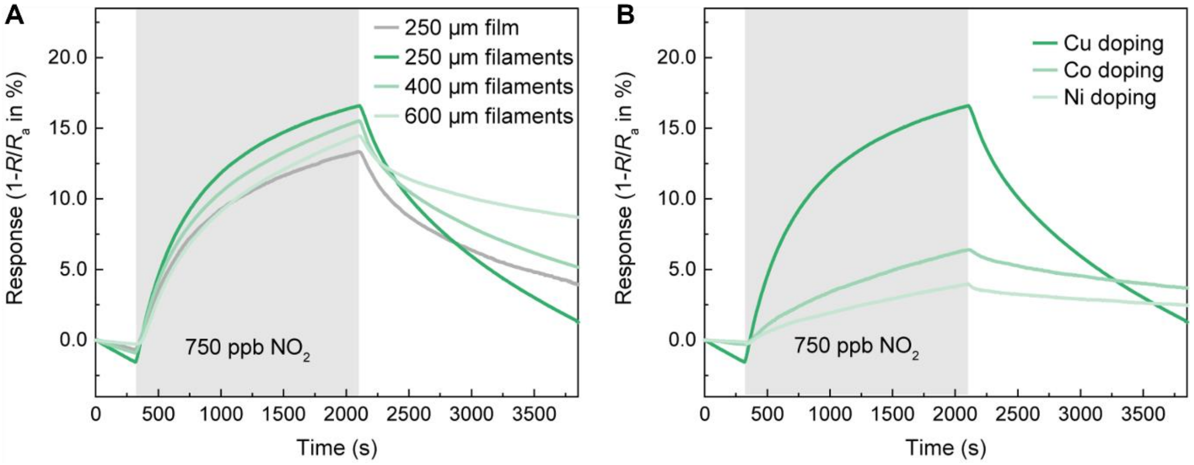

Fig. S12. Room temperature gas sensing characteristics of aerogels towards 750 ppb NO_2.

(**A**) Response curves of filament-structured aerogel sensors with different filament diameters and one film-like aerogel sensor. (**B**) Response curves of filament-structured aerogel sensors with different dopants.

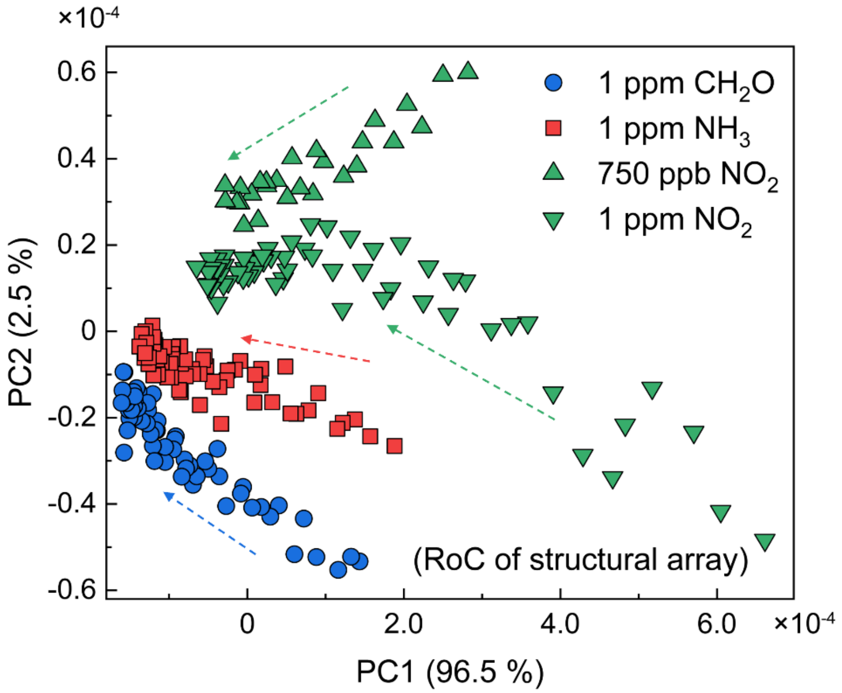

Fig. S13. Principal component analysis (PCA) of RoC features based on aerogel arrays of different structures including thin-film and filaments of different diameters.

The arrows point towards the direction of time progression after exposure to the target gas species.

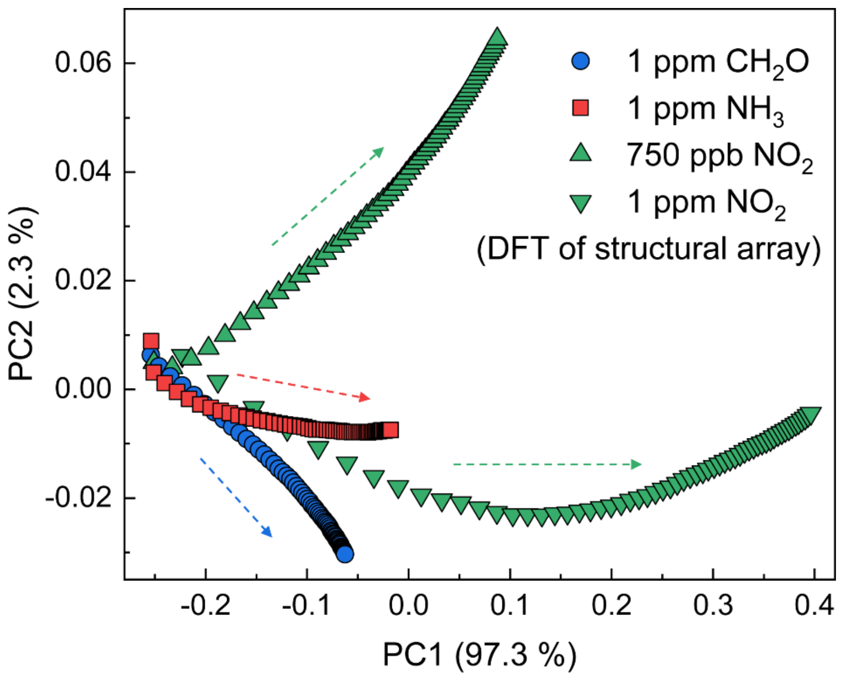

Fig. S14. Principal component analysis (PCA) of DFT features based on aerogel arrays of different structures including thin-film and filaments of different diameters.

This includes the early-stage response data at one to four minutes after exposure to the target gas species. The arrows point towards the direction of time progression.

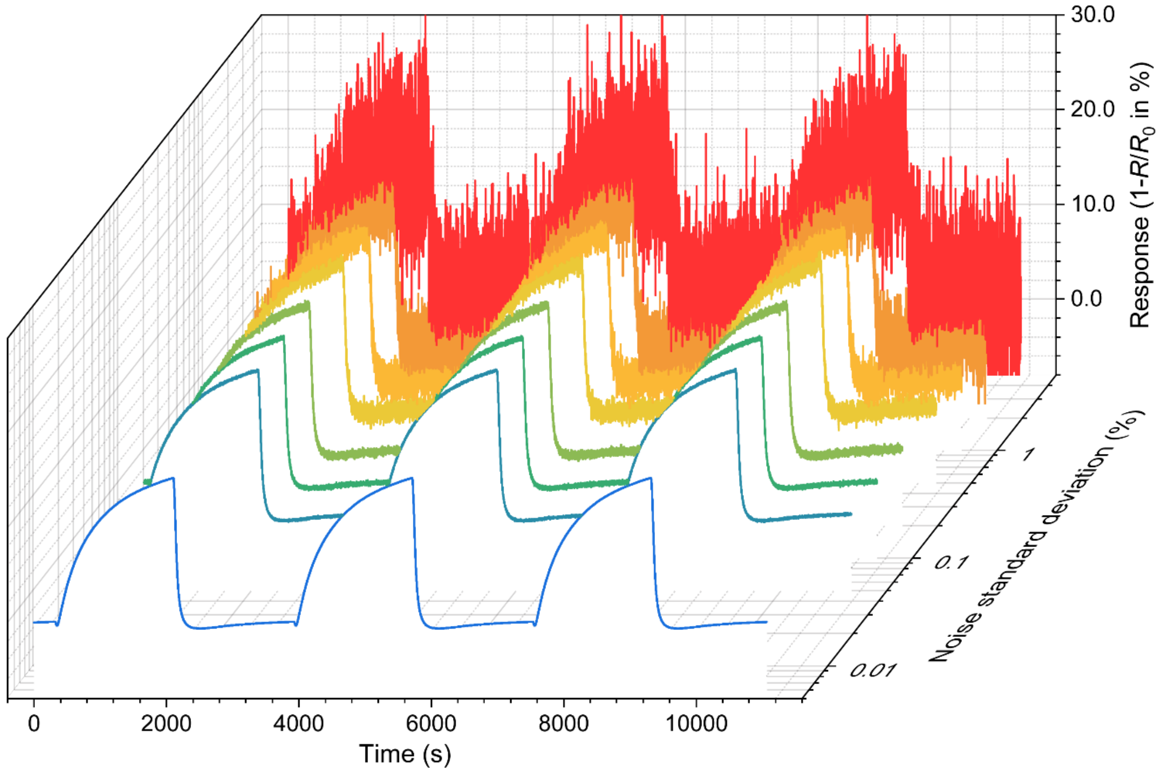

Fig. S15. Response curves with simulated Gaussian noise of standard deviation from 0.05 % to 5 %.

**Algorithms**	**RoC (30s)**	**RoC (PCA)**	**DFT (M_{f0}:M_{f1})**	**DFT(PCA)**
Decision Tree	86.7%	96.3%	91.5%	96.7%
kNN	96.2%	96.7%	98.0%	98.3%
Linear Discriminant	95.0%	89.2%	97.9%	89.6%
Naïve Bayes	63.7%	94.2%	80.4%	96.2%
Support Vector Machine	**96.7%**	**97.5%**	**99.2%**	**99.2%**

Table S1. Comparison of different classifiers' accuracies using dynamic features extracted from the response curves of aerogel arrays of different structures.

The observation window is 30 s, and no simulated noise is added.

Algorithms	RoC (30s)	RoC (PCA)	DFT (M_{f0}:M_{f1})	DFT(PCA)
Decision Tree	77.5%	71.7%	96.3%	92.9%
kNN	84.6%	73.7%	**98.3%**	**99.2%**
Linear Discriminant	81.2%	69.6%	97.1%	68.7%
Naïve Bayes	68.3%	63.8%	83.7%	81.7%
Support Vector Machine	**85.4%**	**74.2%**	**98.3%**	97.8%

Table S2. Comparison of different classifiers' accuracies using dynamic features extracted from the response curves of aerogel arrays of different dopants.

The observation window is 30 s, and no simulated noise is added.

Sensing Materials	Concentration	Response	LOD	Ref.
In_2O_3/MoS_2	1 ppm	6.5 %	200 ppb	*52*
SnO_2/vertical graphene	1 ppm	1.5 %	20 ppb	*53*
SnO_2/ZnO	1 ppm	4.8 %	6 ppb	*54*
ZIF-8/MWCNTs	10 ppm	10 %	4.83 ppm	*55*
Ni-In_2O_3/WS_2	2 ppm	15 %	15 ppb	*56*
ZnO/ANS-rGO	1 ppm	10%	250 ppb	*57*
**SnO_2/rGO aerogels**	**1 ppm**	**15.23 %**	**8 ppb**	**This work**

Table S3. Room-temperature and low-power-consumption CH_2O gas-sensing performance comparison.

Materials with noble metal doping are not included due to concerns about their long-term stability.

Data S1. Surface porosity calculation code. (separate file)

REFERENCES

1. IARC working group on the evaluation of carcinogenic risks to humans, Chemical agents and related occupations (International Agency for Research on Cancer, Lyon, vol.100, 2012).

2. T. Salthammer, S. Mentese, R. Marutzky, Formaldehyde in the indoor environment. *Chem. Rev.* **110**, 2536–2572 (2010).

3. R. Golden, Identifying an indoor air exposure limit for formaldehyde considering both irritation and cancer hazards. *Crit. Rev. Toxicol.* **41**, 672–721 (2011).

4. S. Y. Jeong, J. S. Kim, J. H. Lee, Rational design of semiconductor-based chemiresistors and their libraries for next-generation artificial olfaction. *Adv. Mater.* **32**, e2002075 (2020).

5. J.-H. Lee, Linear gas sensing with dielectric excitation. *Nat. Electron.* **3**, 239–240 (2020).

6. J. Dai, O. Ogbeide, N. Macadam, Q. Sun, W. Yu, Y. Li, B. L. Su, T. Hasan, X. Huang, W. Huang, Printed gas sensors. *Chem. Soc. Rev.* **49**, 1756–1789 (2020).

7. X. Liu, S. Cheng, H. Liu, S. Hu, D. Zhang, H. Ning, A survey on gas sensing technology. *Sensors* **12**, 9635–9665 (2012).

8. M. E. Franke, T. J. Koplin, U. Simon, Metal and metal oxide nanoparticles in chemiresistors: Does the nanoscale matter? *Small* **2**, 36–50 (2006).

9. N. Joshi, T. Hayasaka, Y. Liu, H. Liu, O. N. Oliveira Jr, L. Lin, A review on chemiresistive room temperature gas sensors based on metal oxide nanostructures, graphene and 2D transition metal dichalcogenides. *Microchim. Acta* **185**, 213 (2018).

10. Z. Song, W. Ye, Z. Chen, Z. Chen, M. Li, W. Tang, C. Wang, Z. Wan, S. Poddar, X. Wen, X. Pan, Y. Lin, Q. Zhou, Z. Fan, Wireless self-powered high-performance integrated nanostructured-gas-sensor network for future smart homes. *ACS Nano* **15**, 7659–7667 (2021).

11. Z. Chen, Z. Chen, Z. Song, W. Ye, Z. Fan, Smart gas sensor arrays powered by artificial intelligence. *J. Semicond.* **40**, 111601 (2019).

12. Y. K. Jo, S.-Y. Jeong, Y. K. Moon, Y.-M. Jo, J.-W. Yoon, J.-H. Lee, Exclusive and ultrasensitive detection of formaldehyde at room temperature using a flexible and monolithic chemiresistive sensor. *Nat. Commun.* **12**, 4955 (2021).

13. M. Khatib, H. Haick, Sensors for volatile organic compounds. *ACS Nano* **16**, 7080–7115 (2022).

14. J. Chen, Z. Chen, F. Boussaid, D. Zhang, X. Pan, H. Zhao, A. Bermak, C. Y. Tsui, X. Wang, Z. Fan, Ultra-low-power smart electronic nose system based on three-dimensional tin oxide nanotube arrays. *ACS Nano* **12**, 6079–6088 (2018).

15. A. T. Güntner, V. Koren, K. Chikkadi, M. Righettoni, S. E. Pratsinis, E-nose sensing of low-ppb formaldehyde in gas mixtures at high relative humidity for breath screening of lung cancer? *ACS Sens.* **1**, 528–535 (2016).

16. H. M. Fahad, H. Shiraki, M. Amani, C. Zhang, V. S. Hebbar, W. Gao, H. Ota, M. Hettick, D. Kiriya, Y.-Z. Chen, Y.-L. Chueh, A. Javey, Room temperature multiplexed gas sensing using chemical-sensitive 3.5-nm-thin silicon transistors. *Sci. Adv.* **3**, e1602557 (2017).

17. T.-C. Wu, A. De Luca, Q. Zhong, X. Zhu, O. Ogbeide, D. S. Um, G. Hu, T. Albrow-Owen, F. Udrea, T. Hasan, Inkjet-printed CMOS-integrated graphene–metal oxide sensors for breath analysis. *NPJ 2D Mater. Appl.* **3**, 42 (2019).

18. O. Ogbeide, G. Bae, W. Yu, E. Morrin, Y. Song, W. Song, Y. Li, B.-L. Su, K.-S. An, T. Hasan, Inkjet-printed rGO/binary metal oxide sensor for predictive gas sensing in a mixed environment. *Adv. Funct. Mater.* **32**, 2113348 (2022).

19. W. Tang, Z. Chen, Z. Song, C. Wang, Z. Wan, C. L. J. Chan, Z. Chen, W. Ye, Z. Fan, Microheater integrated nanotube array gas sensor for parts-per-trillion level gas detection and single sensor-based gas discrimination. *ACS Nano* **16**, 10968–10978 (2022).

20. A. C. Romain, J. Nicolas, Long term stability of metal oxide-based gas sensors for e-nose environmental applications: An overview. *Sens. Actuators B* **146**, 502–506 (2010).

21. G. Müller, G. Sberveglieri, Origin of baseline drift in metal oxide gas sensors: Effects of bulk equilibration. *Chem* **10**, 171 (2022).

22. S. Dolai, S. K. Bhunia, R. Jelinek, Carbon-dot-aerogel sensor for aromatic volatile organic compounds. *Sens. Actuators B* **241**, 607–613 (2017).

23. X. Liu, J. Cui, J. Sun, X. Zhang, 3D graphene aerogel-supported SnO_2 nanoparticles for efficient detection of NO_2. *RSC Adv.* **4**, 22601–22605 (2014).

24. Z. Meng, R. M. Stolz, L. Mendecki, K. A. Mirica, Electrically-transduced chemical sensors based on two-dimensional nanomaterials. *Chem. Rev.* **119**, 478–598 (2019).

25. B. Zhou, Z. Chen, Q. Cheng, M. Xiao, G. Bae, D. Liang, T. Hasan, Controlling surface porosity of graphene-based printed aerogels. *NPJ 2D Mater. Appl.* **6**, 34 (2022).

26. J. Feng, B. L. Su, H. Xia, S. Zhao, C. Gao, L. Wang, O. Ogbeide, J. Feng, T. Hasan, Printed aerogels: Chemistry, processing, and applications. *Chem. Soc. Rev.* **50**, 3842–3888 (2021).

27. Health and Safety Executive, EH40/2005 Workplace exposure limits (The Stationery Office, Norwick, ed. 4, 2020).

28. Y. Luo, S. Fan, Y. Luo, N. Hao, S. Zhong, W. Liu, Assembly of SnO_2 quantum dots on RGO to form SnO_2/N doped RGO as a high-capacity anode material for lithium ion batteries. *CrstEngComm* **17**, 1741–1744 (2015).

29. H. Bai, C. Li, X. Wang, G. Shi, On the gelation of graphene oxide. *J. Phys. Chem. C* **115**, 5545–5551 (2011).

30. Z. Song, S. Xu, J. Liu, Z. Hu, N. Gao, J. Zhang, F. Yi, G. Zhang, S. Jiang, H. Liu, Enhanced catalytic activity of SnO_2 quantum dot films employing atomic ligand-exchange strategy for fast response H_2S gas sensors. *Sens. Actuators B* **271**, 147–156 (2018).

31. M. A. Boles, D. Ling, T. Hyeon, D. V. Talapin, The surface science of nanocrystals. *Nat. Mater.* **15**, 141–153 (2016).

32. K. K. H. De Silva, H. H. Huang, M. Yoshimura, Progress of reduction of graphene oxide by ascorbic acid. *Appl. Surf. Sci.* **447**, 338–346 (2018).

33. Z. Sui, X. Zhang, Y. Lei, Y. Luo, Easy and green synthesis of reduced graphite oxide-based hydrogels. *Carbon* **49**, 4314–4321 (2011).

34. H.-J. Kim, J.-H. Lee, Highly sensitive and selective gas sensors using p-type oxide semiconductors: Overview. *Sens. Actuators B* **192**, 607–627 (2014).

35. X. Peng, J. Liu, Y. Tan, R. Mo, Y. Zhang, A CuO thin film type sensor via inkjet printing technology with high reproducibility for ppb-level formaldehyde detection. *Sens. Actuators B* **362**, 131775 (2022).

36. H. Chen, A. Pei, J. Wan, D. Lin, R. Vilá, H. Wang, D. Mackanic, H. G. Steinrück, W. Huang, Y. Li, A. Yang, J. Xie, Y. Wu, H. Wang, Y. Cui, Tortuosity effects in lithium-metal host anodes. *Joule* **4**, 938–952 (2020).

37. K.-W. Choi, J.-S. Lee, M.-H. Seo, M.-S. Jo, J.-Y. Yoo, G. S. Sim, J.-B. Yoon, Batch-fabricated CO gas sensor in large-area (8-inch) with sub-10 mW power operation. *Sens. Actuators B* **289**, 153–159 (2019).

38. I. Cho, Y. C. Sim, M. Cho, Y.-H. Cho, I. Park, Monolithic micro light-emitting diode/metal oxide nanowire gas sensor with microwatt-level power consumption. *ACS Sens.* **5**, 563–570 (2020).

39. G. Sakai, N. Matsunaga, K. Shimanoe, N. Yamazoe, Theory of gas-diffusion controlled sensitivity for thin film semiconductor gas sensor. *Sens. Actuators B* **80**, 125–131 (2001).

40. S. Momeni, F. Sedaghati, CuO/Cu_2O nanoparticles: A simple and green synthesis, characterization and their electrocatalytic performance toward formaldehyde oxidation. *Microchem. J.* **143**, 64–71 (2018).

41. L. Y. Zhu, K. Yuan, J. G. Yang, H. P. Ma, T. Wang, X. M. Ji, J. J. Feng, A. Devi, H. L. Lu, Fabrication of heterostructured p-CuO/n-SnO_2 core-shell nanowires for enhanced sensitive and selective formaldehyde detection. *Sens. Actuators B* **290**, 233–241 (2019).

42. T. C. Wu, J. Dai, G. Hu, W. B. Yu, O. Ogbeide, A. De Luca, X. Huang, B. L. Su, Y. Li, F. Udrea, T. Hasan, Machine-intelligent inkjet-printed α-Fe_2O_3/rGO towards NO_2 quantification in ambient humidity. *Sens. Actuators B* **321**, 128446 (2020).

43. S. Brahim-Belhouari, A. Bermak, P. C. H. Chan, Gas identification with microelectronic gas sensor in presence of drift using robust GMM. *ICASSP, IEEE Int. Conf. Acoust. Speech Signal Process. - Proc.* 5 (2004).

44. A. U. Rehman, A. Bermak, Drift-insensitive features for learning artificial olfaction in e-nose system. *IEEE Sens. J.* **18**, 7173–7182 (2018).

45. E. N. Fuller, K. Ensley, J. C. Giddings, Diffusion of halogenated hydrocarbons in helium. The effect of structure on collision cross sections. *J. Phys. Chem.* **73**, 3679–3685 (1969).

46. B. E. Poling, J. M. Prausnitz, J. P. O'Connell, *Properties of gases and liquids* (McGraw-Hill Educ., New York, ed. 5, 2001).

47. S. Bin Jo, H. J. Kim, J. H. Ahn, B. W. Hwang, J. S. Huh, D. Ragupathy, S. C. Lee, J. C. Kim, Effects of thin-film thickness on sensing properties of SnO_2-based gas sensors for the detection of H_2S gas at ppm levels . *J. Nanosci. Nanotechnol.* **20**, 7169–7174 (2020).

48. S. Di, M. Falasconi, Drift correction methods for gas chemical sensors in artificial olfaction systems: Techniques and challenges. *Adv. Chem. Sensors* (2012).

49. F. A. Akgul, G. Akgul, N. Yildirim, H. E. Unalan, R. Turan, Influence of thermal annealing on microstructural, morphological, optical properties and surface electronic structure of copper oxide thin films. *Mater. Chem. Phys.* **147**, 987–995 (2014).

50. M. Martínez-Gil, D. Cabrera-German, M. I. Pintor-Monroy, J. A. García-Valenzuela, M. Cota-Leal, W. De la Cruz, M. A. Quevedo-Lopez, R. Pérez-Salas, M. Sotelo-Lerma, Effect of annealing temperature on the thermal transformation to cobalt oxide of thin films obtained via chemical solution deposition. *Mater. Sci. Semicond. Process.* **107**, 104825 (2020).

51. A. Liu, G. Liu, H. Zhu, B. Shin, E. Fortunato, R. Martins, F. Shan, Hole mobility modulation of solution-processed nickel oxide thin-film transistor based on high-k dielectric. *Appl. Phys. Lett.* **108**, 233506 (2016).

52. D. Zhang, C. Jiang, J. Wu, Layer-by-layer assembled In_2O_3 nanocubes/flower-like MoS_2 nanofilm for room temperature formaldehyde sensing. *Sens. Actuators B* **273**, 176–184 (2018).

53. Z. Bo, M. Yuan, S. Mao, X. Chen, J. Yan, K. Cen, Decoration of vertical graphene with tin dioxide nanoparticles for highly sensitive room temperature formaldehyde sensing. *Sens. Actuators B* **256**, 1011–1020 (2018).

54. L. Zhao, Y. Chen, X. Li, X. Li, S. Lin, T. Li, M. N. Rumyantseva, A. M. Gaskov, Room temperature formaldehyde sensing of hollow SnO_2/ZnO heterojunctions under UV-LED activation. *IEEE Sens. J.* **19**, 7207–7214 (2019).

55. N. Jafari, S. Zeinali, Highly rapid and sensitive formaldehyde detection at room temperature using a ZIF-8/MWCNT nanocomposite. *ACS Omega.* **5**, 4395–4402 (2020).

56. D. Zhang, Y. Cao, Z. Yang, J. Wu, Nanoheterostructure construction and DFT study of Ni-doped In_2O_3 nanocubes/WS_2 hexagon nanosheets for formaldehyde sensing at room temperature. *ACS Appl. Mater. Interfaces* **12**, 11979–11989 (2020).

57. H. Hu, H. Liang, J. Fan, L. Guo, H. Li, N. F. De Rooij, A. Umar, H. Algarni, Y. Wang, G. Zhou, Assembling hollow cactus-like ZnO nanorods with dipole-modified graphene nanosheets for practical room-temperature formaldehyde sensing. *ACS Appl. Mater. Interfaces* **14**, 13186–13195 (2022).
