# Supplementary material for: Real-time, noise and drift resilient formaldehyde sensing at room temperature with aerogel filaments
Source: Sci Adv. 2024 Feb 9;10(6):eadk6856. doi: 10.1126/sciadv.adk6856 (PMC10857368; doi:10.1126/sciadv.adk6856)
Supplement: Supplementary file 2 — Data S1 [file sciadv.adk6856_data_s1.zip › adk6856_Data_S1.pdf]

**Data S1. Surface porosity calculation code.**

```
% Surface Porosity Calculation
% Need image processing license
% By Zhuo Chen & Binghan Zhou
% zc326@cam.ac.uk

% Optimised from following reference:
% Rabbani, A., Ayatollahi, S. (2015). Comparing three image processing
% algorithms to estimate the grain-size distribution of porous rocks
% from binary 2d images and sensitivity analysis of the grain overlapping
% degree. Special Topics & Reviews in Porous Media: An International
% Journal 6 (1), 71-89.

clc;
clear;
close all;

% Input SEM image
imagefiles = dir('*.tif');
nfiles = length(imagefiles);    % Number of files found
for ii=1:nfiles
   currentfilename = imagefiles(ii).name;
   [x, y] = size(currentfilename);
   currentfilenameText = currentfilename(1:(y-4));
   currentImage = imread(currentfilename);
   images{ii} = currentImage;
   [rows, columns, numberOfColorChannels] = size(currentImage);

   % Background estimation and removal for non-uniform illumination.
   i_1_backgroud = imopen(images{1,ii},strel('square',200));
   % 200 or less for a normal image. Enlarge the number to 500 or larger
   % if the illumination is extremely uneven.
   imwrite(i_1_backgroud,'1-Background.jpg');
   i_2_substrated = imsubtract(images{1,ii},i_1_backgroud);
   imwrite(i_2_substrated,'2-Substracted Background.jpg');

   % Binarise the figure
   if numberOfColorChannels == 1
       i_3_bi = i_2_substrated;
   else
       i_3_bi = rgb2gray(i_2_substrated);
   end
   i_3_bi = imadjust(i_3_bi,[0, 1],[0, 1]);
   % Modulate contrast for better fitting
   imwrite(i_3_bi ,'3-Contrast Adjustment.jpg');
   % Contrast adjustment results
   T = adaptthresh(i_3_bi, 0.5);    % Sensitivity of binarisation
   i_3_bi = imbinarise(i_3_bi, T);
   imwrite(i_3_bi,'3-Binary.jpg');
   i_3_re = ~i_3_bi;    % Reverse image
```

```
    imwrite(~i_3_re,[currentfilenameText '-reverse.bmp']);


    % Match holes/porous area.
    i_4_holes = imfill(i_3_re,'holes');
    imwrite(i_4_holes,[currentfilenameText '-Holes.bmp']);

    % Overlay original image and create boundary for reference.
    i_5_overlay=labeloverlay(images{1,ii},i_4_holes,'Colormap', jet(1));
    imwrite(i_5_overlay,[currentfilenameText '-Overlaid.bmp']);
    i_5_outline = bwperim(i_4_holes);

    % Ignore the bottom part of SEM figure.
    actual_rows = round(1024/1103*rows);
    i_4_holes_actual=i_4_holes(1:actual_rows,:);

    % Calculation the surface porosity.
    if currentfilenameText(1)=='+'
        SurfacePorosity=sum(i_4_holes,'all')/(rows*columns);
    else
        SurfacePorosity=sum(i_4_holes_actual,'all')/(actual_rows*columns);
    end

    porosityTable(ii,1)=convertCharsToStrings(currentfilenameText);
    porosityTable(ii,2)=SurfacePorosity;
end
```
